# High risk groups for severe COVID-19 in a whole of population cohort in Australia

**DOI:** 10.1186/s12879-021-06378-z

**Published:** 2021-07-16

**Authors:** Bette Liu, Paula Spokes, Wenqiang He, John Kaldor

**Affiliations:** 1 School of Population Health, UNSW, Sydney, Australia; 2 Public Health Response Branch, NSW Ministry of Health, St. Leonards, Australia; 3 Kirby Institute, UNSW, Sydney, Australia

## Abstract

**Background:**

Increasing age is the strongest known risk factor for severe COVID-19 disease but information on other factors is more limited.

**Methods:**

All cases of COVID-19 diagnosed from January–October 2020 in New South Wales Australia were followed for COVID-19-related hospitalisations, intensive care unit (ICU) admissions and deaths through record linkage. Adjusted hazard ratios (aHR) for severe COVID-19 disease, measured by hospitalisation or death, or very severe COVID-19, measured by ICU admission or death according to age, sex, socioeconomic status and co-morbidities were estimated.

**Results:**

Of 4054 confirmed cases, 468 (11.5%) were classified as having severe COVID-19 and 190 (4.7%) as having very severe disease. After adjusting for sex, socioeconomic status and comorbidities, increasing age led to the greatest risk of very severe disease. Compared to those 30–39 years, the aHR for ICU or death from COVID-19 was 4.45 in those 70–79 years; 8.43 in those 80–89 years; 16.19 in those 90+ years. After age, relative risks for very severe disease associated with other factors were more moderate: males vs females aHR 1.40 (95%CI 1.04–1.88); immunosuppressive conditions vs none aHR 2.20 (1.35–3.57); diabetes vs none aHR 1.88 (1.33–2.67); chronic lung disease vs none aHR 1.68 (1.18–2.38); obesity vs not obese aHR 1.52 (1.05–2.21). More comorbidities was associated with significantly greater risk; comparing those with 3+ comorbidities to those with none, aHR 5.34 (3.15–9.04).

**Conclusions:**

In a setting with high COVID-19 case ascertainment and almost complete case follow-up, we found the risk of very severe disease varies by age, sex and presence of comorbidities. This variation should be considered in targeting prevention strategies.

## Key points

- The risk of severe COVID-19 increases exponentially with increasing age.
- A single comorbidity increased the risk of severe COVID-19 moderately (2-fold increase).
- However multimorbidity (3 or more comorbidities) led to a 5-fold higher risk of severe COVID-19.

## Introduction

Since SARS-CoV-2 emerged in late 2019 efforts have been underway to understand the epidemiology of both the infection and the disease it causes, COVID-19, in order to reduce its health impacts. Public health measures including isolation of cases and contacts as well as population-wide restrictions on movement have been effective to varying degrees but ultimately vaccination is the most feasible long-term strategy for control.

Identifying and quantifying the high-risk groups for COVID-19 is a key consideration in prevention strategies including the design of equitable and effective vaccination programs. High-risk groups can be defined as both those at higher likelihood of contracting and transmitting the infection such as health and aged care workers, and those for whom the infection, if acquired, has a higher likelihood of causing severe disease or death.

Increasing age has consistently been demonstrated to strongly increase the likelihood of severe disease and death from COVID-19 [1] but information on other characteristics has been less clear [2]. Factors identified with varying degrees of consistency include male sex, lower socioeconomic status, ethnicity, smoking and certain co-morbidities including hypertension, cardiovascular disease, chronic lung disease, diabetes, and obesity [2]. A meta-analysis of 77 studies with more than 38,000 patients hospitalised with COVID-19 reported that hypertension was associated with an increased relative risk of death (RR 1.76) with significant heterogeneity among studies (I^2^ = 57%, *P* < 0.01) [2]. In contrast, a study from a large electronic primary care database of 17 million people in the UK reported lower risks of death from COVID-19 among those with hypertension (aHR 0.89) [3]. Possible explanations for these differences across studies include the study sampling frame used, and the extent to which confounders were adjusted for [4].

In New South Wales (NSW), an Australian state which includes a third of the population, there has been an aggressive control strategy for SARS-CoV-2 infection, based on very high testing levels and contact tracing [5]. This strategy, combined with the low positivity (generally under 0.05%, as compared to 5–10% in many other countries [6, 7]) since the beginning of the pandemic suggests that most cases are diagnosed. In this setting we have followed virtually all diagnosed COVID-19 cases to identify those with illness requiring hospitalisation, intensive care unit (ICU) admission or resulting in death and report on risk factors for severe COVID-19.

## Methods

### Population and data sources

We used a database recording all people tested for COVID-19 in NSW (the Notifiable Conditions Information Management System (NCIMS)) probabilistically linked to all hospitalisations (NSW Admitted Patient Data Collection; APDC) and death registry records in NSW. These data have been described previously [8]. Briefly the NCIMS is a statutory infectious diseases register that includes records all people tested for COVID-19 in NSW. Data recorded include sociodemographic details as well as the test type, date and result. For cases confirmed as positive according to nationally agreed case definitions [9], additional information is collected via interview regarding symptoms, hospitalisation and comorbidities. The NSW APDC records all inpatient care in NSW and includes coded information on the diagnosis, admission and discharge dates, and admission to ICU. The Death Registry includes information on fact and date of death. All deaths in people with a diagnosis of COVID-19 are assessed to determine if the death was caused by COVID-19 and this outcome is recorded in the NCIMS. For this study we had accessed records from NCIMS, NSW APDC, and the Death Registry to 5 October 2020.

We defined a retrospective cohort consisting of all people diagnosed with COVID-19 in NSW identified from the NCIMS between 1 January and 5 October 2020. “Severe disease” was a composite outcome of either hospitalisation or death from COVID-19 and “very severe disease” was either an ICU admission or death from COVID-19; therefore the “very severe disease” group was a subset of the “severe disease” group. As described previously [8], COVID-19 related hospitalisations and ICU admissions were identified based on linking a case with COVID-19 from NCIMS to a hospital or ICU admission in the 6-week period following the COVID-19 diagnosis. Only in-patient stays of at least one night in hospital were included. We also supplemented the linked records with information on hospitalisations in private hospitals from what was recorded in NCIMS as these may not have been captured in the linked data. COVID-19 related deaths were obtained from the NCIMS record.

Information on sociodemographic characteristics, smoking, pregnancy and selected co-morbidities was obtained from the NCIMS based on the initial interview and subsequent case interviews. Additional comorbidity information was obtained via linkage to hospitalisation records prior to the COVID-19 diagnosis, using the International Classification of Diseases Australian Modification 10 (ICD-10-AM) diagnosis code for specific illnesses. This study was approved by the UNSW Human Research Ethics Committee (HC200483). All methods were performed in accordance with relevant guidelines and regulations.

### Analysis

People were excluded from analyses if they had a missing hospital admission date or if they were already in hospital (for an alternate diagnosis) at the time of COVID-19 onset and did not have a subsequent COVID-19 admission record. For ICU analyses, those reported as admitted to ICU but with a missing ICU admission date, were classified as admitted to ICU on their hospital admission date. People were followed from the date of COVID-19 onset as reported in NCIMS to the first COVID-19 hospitalisation, ICU admission, death or otherwise the end of follow-up which was 42 days following onset; or 5 October 2020 whichever came first. Cox proportional hazards models were used to estimate risks of COVID-19 hospitalisation or death according to age (10-year intervals), sex, socioeconomic status (in deciles using an area-based index [10]), residential classification (in 3 categories: major city, inner regional, outer regional or remote). Comorbidities based only on records from the APDC included: ischaemic heart disease (ICD-10-AM I20-I25); cerebrovascular disease (I60-I69); cancer (excluding non-melanomatous skin cancers) in the year prior to COVID-19 diagnosis (C00-C97, excluding C44); chronic kidney disease (N18). Those based on either a report in the APDC or NCIMS were: hypertension (I10); chronic obstructive pulmonary disease or bronchitis (J40-J44); asthma (J45); obesity (E66); diabetes (E10-E14); an immunosuppressive condition (which included hospitalisation with organ transplant, haematopoietic cancer, rheumatoid arthritis, Crohn’s disease or ulcerative colitis – ICD-10-AM C81-C96, K50-K51, M05-M06, Y83.0, Z94). Information on smoking or pregnancy was based on NCIMS. In addition, variables encompassing, “any comorbidity” and the total number of comorbidities (0, 1, 2 or 3+) were created based on a diagnosis of any of the conditions listed above except smoking and pregnancy.

Age- and sex-adjusted rate ratios were calculated and then further adjusted for socioeconomic status, and presence of “any comorbidity”. Analyses were repeated with the outcome of “very severe COVID-19”. Sensitivity analyses were conducted by restricting analyses to those who acquired infection locally; and including only cases where their onset date was after 1 March 2020 to account for the practice in the early weeks of the pandemic of admitting all cases to hospital for observation and monitoring rather than due to clinical need [8].

## Results

From 1 January to 5 October 2020 there were 4055 confirmed cases of COVID-19 diagnosed in NSW; their mean age was 44.7 years (SD 20.2), 49.8% (*n* = 2019) were female, 84.2% (*n* = 3413) were resident in a major city and 34.7% (*n* = 1406) were classified in the lower 50% of socioeconomic deciles (7.2%, *n* = 290, were missing socioeconomic classification).

Of the 4055 confirmed cases, one was excluded from analyses (due to missing hospitalisation date). Of the remaining 4054 cases, 447 were admitted to hospital as inpatients following COVID-19 onset, of whom 34 died from COVID-19. Another 21, mostly from residential aged care facilities, were recorded as having died from COVID-19 but were not admitted to hospital, so in total 468 were classified as having a severe COVID-19 outcome (hospitalisation and/or death). Of the 468, 190 were either admitted to ICU or died and classified as having very severe COVID-19 outcomes.

**Fig. 1:**
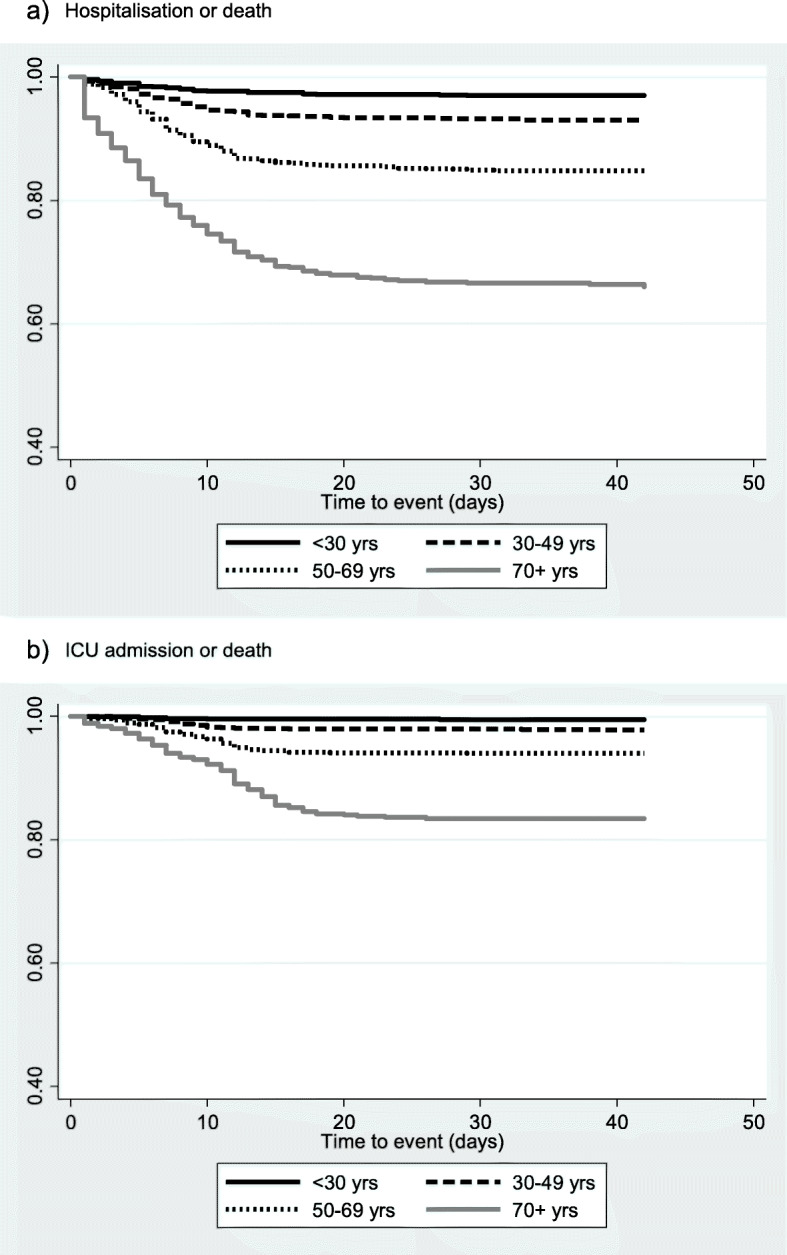
Kaplan Meier survival curve for time to **a** hospitisation or death and **b** ICU admission or death according to age

As shown in Fig. 1 for a) severe COVID-19 and b) very severe COVID-19 there is a clear increase in the incidence of poor COVID-19 outcomes with age, with adults 70+ years much more likely to be hospitalised, admitted to ICU or to die from COVID-19.

**Fig. 2:**
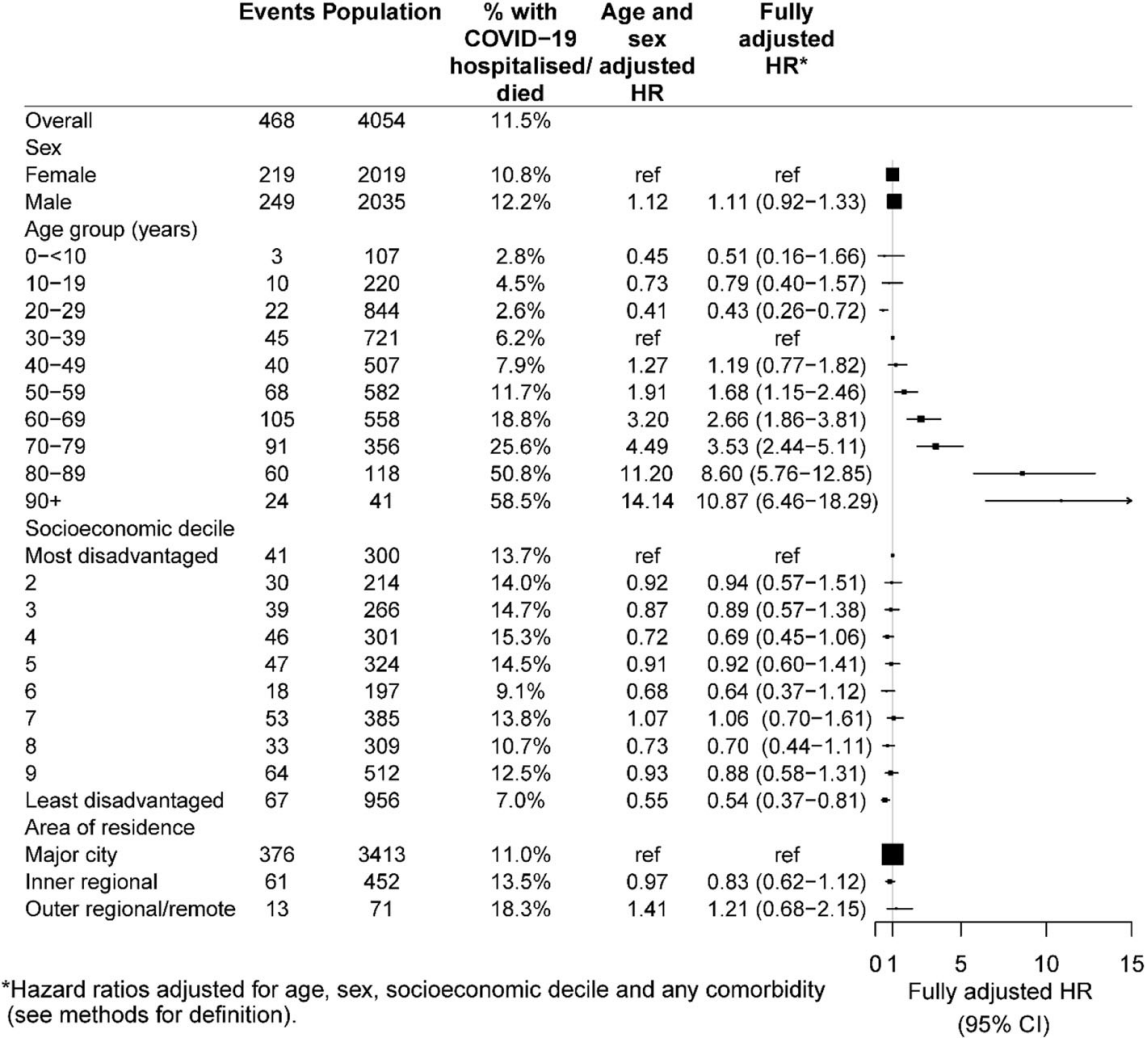
Hazard ratios for risk of severe COVID-19 (hospitalisation or death) according to age and other sociodemographic characteristics

Figure 2 shows the proportion of COVID-19 cases classified as severe by age, sex, socioeconomic status and area of residence, as well as the adjusted hazard ratios. Among adults, increasing age was the characteristic most strongly associated with severe COVID-19. After adjustments, compared to those aged 30–39 years the risk of severe COVID-19 was almost 10 times greater in those aged 80+ years and in those aged 60–79 years it was about 3 times higher. There was no significant effect of sex, with men and women having similar risk of COVID-19 hospitalisation or death (aHR 1.11, 95%CI 0.92–1.33). There was also a strong effect, only in the most socioeconomically advantaged group of reduced risk of severe COVID-19 (aHR 0.54, 95%CI 0.37–0.81).

**Fig. 3:**
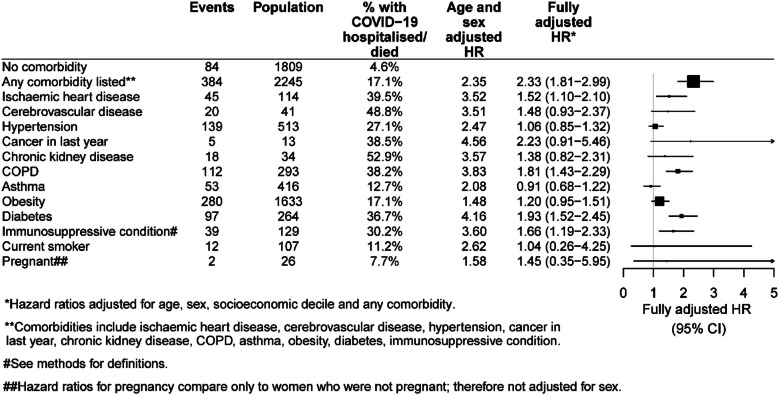
Hazard ratios for risk of severe COVID-19 (hospitalisation or death) according to co-morbidities and other factors compared to those without each comorbidity

Figure 3 shows the numbers and proportions of cases with severe COVID-19 according to comorbidities, and the adjusted hazard ratios comparing those with the comorbidity to those without. Of those without a comorbidity, around 5% were hospitalised or died. This compares to 49% and 53% in those with cerebrovascular disease or chronic kidney disease. However after adjusting for age, sex, socioeconomic status and the presence of other comorbidities, these risks were attenuated. In fully adjusted models, the highest hazard ratios for a single comorbidity were in people with diabetes, aHR 1.93 (95%CI 1.52–2.45) compared to those without diabetes, with COPD/bronchitis compared to those without, aHR 1.81 (95%CI 1.43–2.29), and for those with immunosuppressive conditions aHR 1.66 (95%CI 1.19–2.33) compared to those without. Having any of the examined comorbidities compared to none was also associated with increased risk with aHRs of 2.33 (95%CI 1.81–2.99). Neither being a current smoker or pregnancy was found to be associated with increased risk of hospitalisation or death from COVID-19 although the numbers of pregnant women diagnosed with COVID-19 was relatively small.

**Fig. 4:**
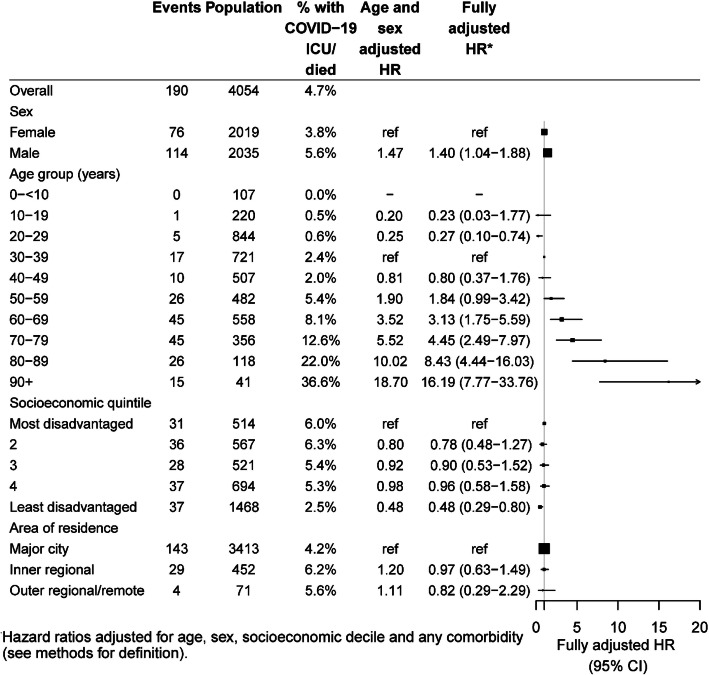
Hazard ratios for risk of very severe COVID-19 (ICU admission or death) according to sociodemographic characteristics

**Fig. 5:**
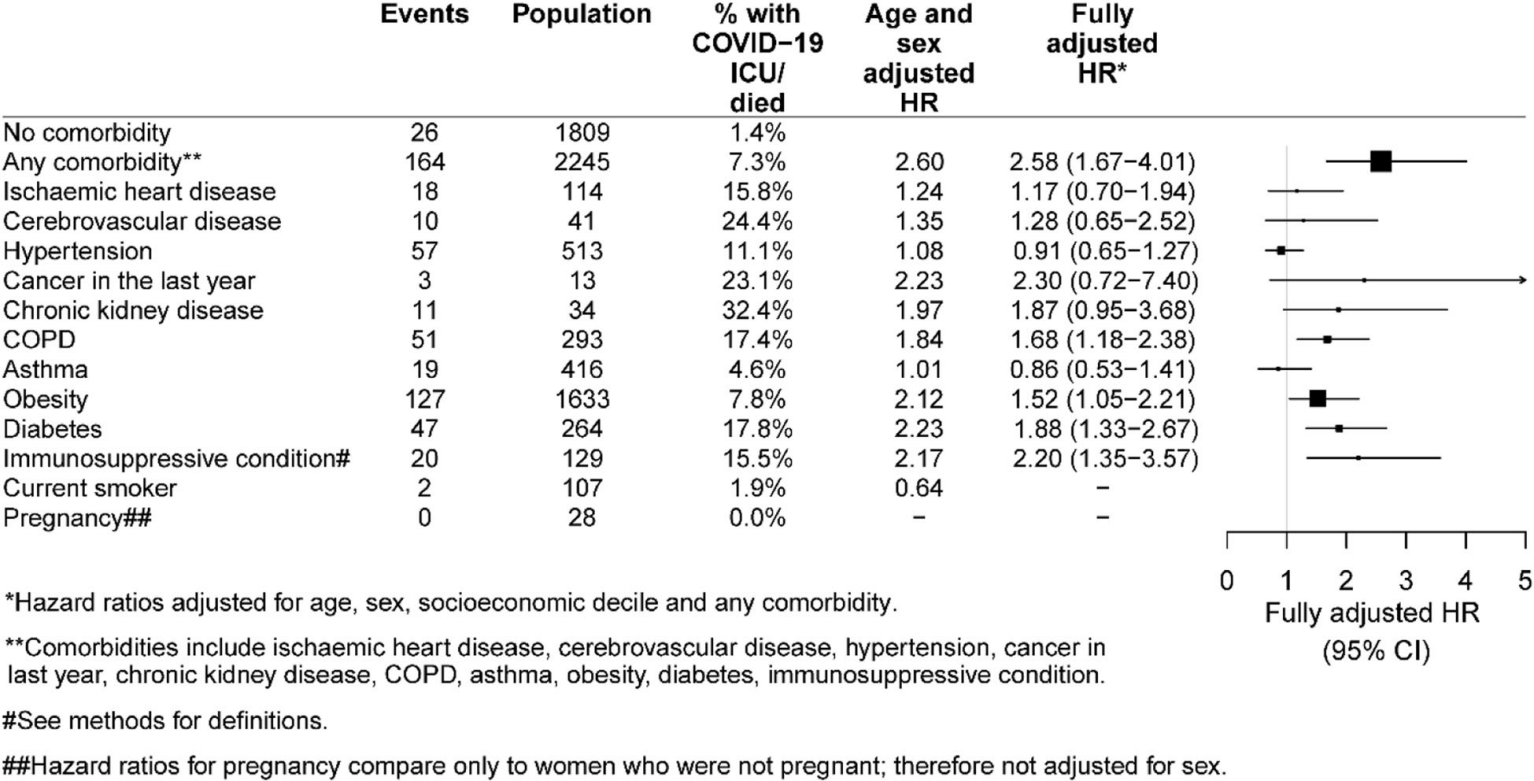
Hazard ratios for risk of very severe COVID-19 (ICU admission or death) according to co-morbidities and other factors compared to those without each comorbidity

Figures 4 and 5 show hazard ratios for very severe COVID-19 (ICU admission or death) based on 190 events among 4054 cases. Results were somewhat similar to those shown in Figs. 2 and 3 examining hospitalisation or death. Older age remained by far the predominant risk factor. Hazard ratios comparing those aged 20–29 to those aged 30–39 years were 0.27 (95%CI 0.10–0.74). Hazard ratios then increased exponentially to 4.45 (95%CI 2.49–7.97) in those 70–79, 8.43 (95%CI 4.44–16.03) in those aged 80–89 years and 16.19 (95%CI 7.77–33.76) in those 90+ years. Men had higher risks for very severe COVID-19 than women (aHR 1.40, 95%CI 1.04–1.88). Also, those who were the least disadvantaged had only half the risk of very severe COVID-19 as those who were most disadvantaged (aHR 0.48, 95%CI 0.29–0.80). For ICU or death from COVID-19, diabetes, immunosuppressive conditions, obesity, and COPD/chronic bronchitis, had the strongest associations. A recent cancer diagnosis and chronic kidney disease were also found to have elevated point estimates for risk of the same magnitude as the other conditions, but numbers were relatively small and fully adjusted hazard ratios were not statistically significant. There were insufficient numbers of smokers and pregnant women who were admitted to ICU or who died from COVID-19 to calculate risk ratios.

**Fig. 6:**
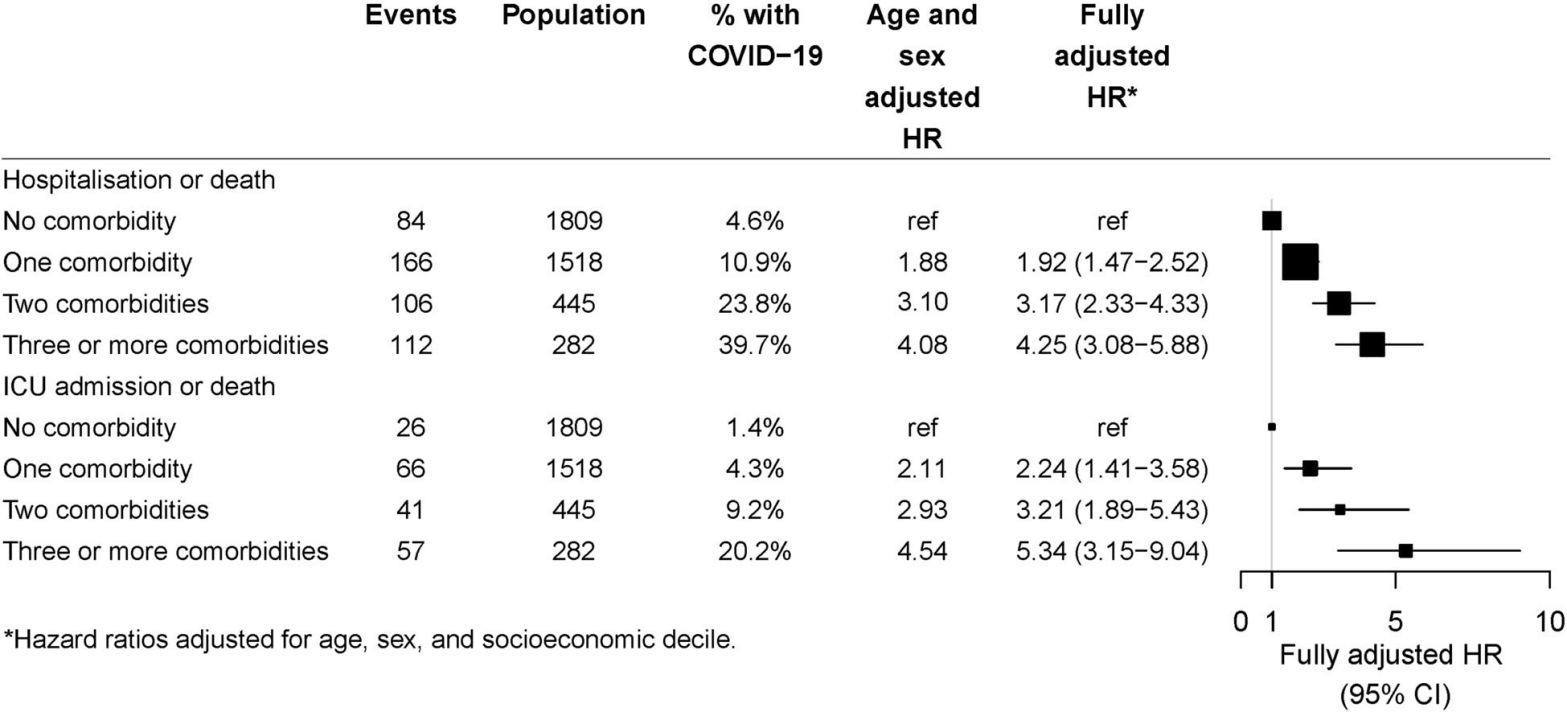
Hazard ratios for risk of severe (hospitalisation or death) and very severe (ICU or death) COVID-19 according to number of comorbidities

Figure 6 shows that risks of both severe and very severe COVID-19 increased significantly with the number of comorbidities. Compared to those without any comorbidities, risks increased linearly with increasing numbers of comorbidities to about 4–5 times higher in those with 3 or more comorbidities.

In sensitivity analyses in which the population was restricted to cases who acquired their infections locally (*N* = 1795) there were no significant deviations from the main findings although for some groups the numbers of cases and events were substantially smaller (data not shown). When we restricted analyses to cases who had an onset date from 1 March 2020, the majority of cases were included in analyses (4004/4054, 99%) so findings were unchanged.

## Discussion

The strengths of this analysis compared to other reports is the inclusion of all cases of COVID-19 diagnosed in a setting that has had high testing, low positivity and robust contact tracing throughout the COVID-19 pandemic. This means that the vast majority of those with COVID-19 are likely to have been included in the study. Additionally, use of record linkage with comprehensive hospital databases means that follow up for severe outcomes is near complete and unbiased. Our study design contrasts with other reports in settings with substantially higher case numbers. The UK OpenSafely study of 17 million adults used record linkage to over 10,000 COVID-19 related deaths to investigate risk factors but did not distinguish those in the cohort who had tested positive for COVID-19 from those who were not tested and so cannot differentiate between risk factors for acquisition versus those for severity of disease once infected [3]. Two recent large meta-analyses of 77 studies and 38,000 cases, and of 61 studies and 31,000 cases, have mostly included only patients already hospitalised with COVID-19 [2, 11]; some of the studies included in the reviews had limited adjustment for potential confounders including age, sex and other comorbidities and the inclusion of only hospitalised cases may bias the findings away from populations such as those in aged care who may not be hospitalised but still experience severe outcomes. Our findings are based on a whole of population sample and include a wide spectrum of people with confirmed COVID-19 diagnoses, the vast majority of whom were not hospitalised for the disease but may regardless have underlying co-morbidities. Therefore our findings, while in a much smaller sample than many other studies internationally are far more generalisable.

Despite the difference in sampling frames compared to reports from other countries, reassuringly our findings regarding high risk groups are mostly consistent. Increasing age far outweighed any other population-based risk factor for severe COVID-19. Compared to people aged 30–39 years, we found that risks of both severe COVID-19 and very severe COVID-19 increased exponentially and were more than 10 times greater in those aged 80 years and above. Risk ratios for specific comorbidities were more moderate. After adjusting for age, sex and, socioeconomic status, no single comorbidity led to a more than doubling of risk; diabetes, COPD, and immunosuppressive conditions were estimated to have the highest risks although people with conditions like a cancer diagnosis in the last year and chronic kidney disease, had relative risks point estimates of around 2, but possibly due to smaller numbers of events, these risk ratios were not statistically significant. Obesity, defined as a BMI 30 + kg/m^2^ was also found to be associated with an increased risk of ICU admission or death from COVID-19. Our estimates of the magnitude of the risks associated with most comorbidities examined are very consistent with large studies from the UK [3] and US [12] that have had careful adjustment for potential confounders. Also similar to the large UK study we did not find that hypertension or smoking conferred higher risks of severe infection, particularly once other comorbidities were considered although we only had data on current smoking and so the comparator group included both past and non-smokers which is likely to attenuate any effects. Fewer studies have reported on risks associated with multiple comorbidities [13]. After adjusting for age, sex and socioeconomic status we found risk ratios for both severe and very severe COVID-19 outcomes increased substantially with those with at least 3 co-morbidities having 4–5 times the risk of those without any.

We found that compared to women, men had about a 40% greater risk of ICU admission or death from COVID-19. This is consistent with most other studies that have adjusted for comorbidities with estimates comparing men to women between 1.5 [12] to 1.76 [14]. In our analyses we did not find that additional adjustment for comorbidities substantially attenuated the relative excess risk of ICU or death from COVID-19 in men compared to women (HR 1.48 adjusted for age; HR 1.42 adjusted for age, socioeconomic decile, area of residence and comorbidities) suggesting, as others have shown, that differences between men and women in their COVID-19 outcomes may be due to other intrinsic factors such their immune responses to infection [15] rather than an underlying susceptibility due to greater likelihood of comorbidities.

The large UK study reported that there was a clear gradient of decreasing risk of COVID-19 death with increasing advantage [3] but could not distinguish whether this was due to a lower risk of acquisition of infection. Another study in the US from a health insurance database found that among adults diagnosed with COVID-19, socioeconomic advantage, based on median income, was associated with less likelihood of hospitalisation but there was no clear gradient [16]. Our analysis, which, similar to the US study, only considered those diagnosed with COVID-19, found that the least disadvantaged group was half as likely to be admitted to ICU or die from COVID-19 than the most disadvantaged; a similar pattern was also found with hospitalisation for COVID-19. Australia has a universal healthcare system and both COVID-19 testing and treatment are free for all people. While our measure of socioeconomic disadvantage is an area-based rather than individual-based measure, this finding suggests either a potential difference in access to treatment or for uncontrolled confounding.

Our study limitations include the relatively small sample size compared to studies from other countries which limits the statistical power to examine some comorbid conditions reliably. With all observational studies there is also the potential for inadequate control of confounders but this limitation in most circumstances is likely to result in over- rather than under-estimating risks associated with comorbid conditions.

Our findings in a whole of population sample with near complete follow-up for severe outcomes from COVID-19 add to the worldwide literature on identifying high risk groups. Vaccination programs are prioritising both those with a higher likelihood of acquiring COVID-19 and transmitting this to others, as well as those who are more likely to experience greater severity of disease if infected [17]. Our results, along with others, clearly point to age as a key consideration for groups at highest risk of severe COVID-19, with smaller relative risks associated with comorbidities once age is considered. Further investigation of the mechanisms behind why some groups, for example men, and those with certain comorbidities have significantly greater risk of higher morbidity associated with COVID-19 is needed. Also, in a setting such as Australia where universal health care is available, further understanding of the reduced risk of severe disease in the least socioeconomically disadvantaged is required.

## Data Availability

The data that support the findings in this study are available from the NSW Ministry of Health but restrictions apply so they are not publicly available. However they could be obtained from the NSW Ministry of Health if an appropriate request was made and ethics approvals obtained for their use.
